# Aberrant *DNMT3B7* expression correlates to tissue type, stage, and survival across cancers

**DOI:** 10.1371/journal.pone.0201522

**Published:** 2018-08-02

**Authors:** Safia Siddiqui, Michael W. White, Aimee M. Schroeder, Nicholas V. DeLuca, Andrew L. Leszczynski, Stacey L. Raimondi

**Affiliations:** Department of Biology, Elmhurst College, Elmhurst, Illinois, United States of America; University of South Alabama Mitchell Cancer Institute, UNITED STATES

## Abstract

Cancer cells are known for aberrant methylation patterns leading to altered gene expression and tumor progression. DNA methyltransferases (DNMTs) are responsible for regulating DNA methylation in normal cells. However, many aberrant versions of DNMTs have been identified to date and their role in cancer continues to be elucidated. It has been previously shown that an aberrant version of a *de novo* methylase, DNMT3B7, is expressed in many cancer cell lines and has a functional role in the progression of breast cancer, neuroblastoma, and lymphoma. It is clear that DNMT3B7 is important to tumor development *in vitro* and *in vivo*, but it is unknown if expression of the transcript in all of these cell lines translates to relevant clinical results. In this study, a bioinformatics approach was utilized to test the hypothesis that *DNMT3B7* expression corresponds to tumor progression in patient samples across cancer types. Gene expression and clinical data were obtained from the Genomic Data Commons for the 33 cancer types available and analyzed for *DNMT3B7* expression with relation to tissue type in matched and unmatched samples, staging of tumors, and patient survival. Here we present the results of this analysis indicating a role for *DNMT3B7* in tumor progression of many additional cancer types. Based on these data, future *in vitro* and *in vivo* studies can be prioritized to examine *DNMT3B7* in cancer and, hopefully, develop novel therapeutics to target this aberrant transcript across multiple tumor types.

## Introduction

The American Cancer Society estimates that nearly 1 out of every 3 people will be diagnosed with cancer in their lifetime [1]. While treatments have significantly improved and patient survival has increased in the last decade, cancer continues to be a global health issue and improved targeted therapies are needed. However, due to the heterogeneity of tumors, it has been difficult to identify one gene or protein that could be targeted to improve treatments across multiple cancer types.

It has been well-documented that cancer cells are characterized by abnormal DNA methylation patterns that alter gene expression and function [2, 3]. Tumor suppressor genes are often hypermethylated and transcriptionally inactive while oncogenes are hypomethylated and active. Normal methylation is regulated by three DNA methyltransferases (DNMTs)–DNMT1, DNMT3A, and DNMT3B [4–6]. DNMT1 is a maintenance methylase that is active throughout life while DNMT3A and DNMT3B are *de novo* methylases that are normally active in early development.

Recently, it has been shown that aberrant versions of DNMT3B are expressed in cancer cells, but not normal cells, and their functional role is still being elucidated [7–11]. Specifically, one of these aberrant transcripts, *DNMT3B7*, is expressed in 21 out of 25 cancer cell lines tested, including both solid and hematopoietic malignancies, making it a novel target that could potentially be used to treat many cancers at once [7]. *DNMT3B7* retains 94bp of intron 10 sequence leading to an early stop codon and truncated protein. Furthermore, this truncated protein retains functional activity as observed by the fact that cell lines stably expressing DNMT3B7 show altered methylation patterns [7]. Shah and colleagues were the first to show that increased DNMT3B7 expression promotes lymphomagenesis in mice and alters methylation patterns *in vivo* as well as *in vitro* [9]. Subsequently, our laboratory has shown that expression of DNMT3B7 promotes tumor progression in breast cancer cells leading to hypermethylation of E-cadherin and corresponding changes in cell adhesion, proliferation, and anchorage-independent growth [10]. Interestingly, expression of DNMT3B7 in neuroblastoma showed an opposing effect in that lower levels of the transcript corresponded to tumor progression as measured by increased cell proliferation, angiogenesis, and tumor formation [11]. It is possible that differences in DNMT3B7 function may be related to cell type, such as changes between epithelial and mesenchymal cells, but additional studies are needed.

Because *DNMT3B7* is expressed in so many different cancer cell types, and retains an intron sequence not found in other genes, it is an attractive target for novel targeted therapies. However, while we know that *DNMT3B7* is expressed in multiple cancer cell lines, it is unknown whether this altered expression is observed in clinical samples. Furthermore, in order to elucidate the role of *DNMT3B7* across all cancer types, *in vitro* and *in vivo* studies are required. Studies of this size and nature are both time-consuming and costly, therefore our laboratory utilized a bioinformatics approach to test the hypothesis that *DNMT3B7* expression promotes tumor progression across cancers as measured by expression in normal versus tumor tissues, staging, and patient survival. The results of this study provide useful information on which cancer types should be further examined *in vivo* with the ultimate goal of developing novel therapeutics to target this aberrant transcript and potentially treat many different cancer types.

## Materials & methods

### Collection of data from Genomic Data Commons

RNAseqV2 and clinical data were obtained from the Genomic Data Commons (GDC) Legacy data portal (https://portal.gdc.cancer.gov/legacy-archive/search/f) [12]. Data were organized and processed using a custom C# script and Microsoft Excel (Redmond, Washington) to analyze expression of the retained 94bp sequence of intron 10 that is specific to *DNMT3B7*. Analyses were conducted on all available patient data for every cancer type available. Clinical staging was measured as stage I (combination of stage I, stage IA, and stage IB), stage II (combination of stage II, stage IIA, stage IIB, and stage IIC), stage III (combination of stage IIIA, stage IIIB, and stage IIIC), or stage IV. In order to determine survival rates, the median of *DNMT3B7* expression across all tumor samples at a given site was determined. The samples were then divided in half, based on the median, into “low” and “high” expression groups and compared [13].

### Statistical analysis

All statistical analysis was performed using SigmaStat software (Systat, Chicago, IL). Tumor versus normal tissue expression was compared using a Student’s T-test while matched tissues were compared with a Pairwise T-Test. Comparisons among groups for staging were analyzed with a one-way ANOVA with Dunn’s multiple comparisons. Finally, Survival LogRank analysis was utilized to generate a Kaplan-Meier curve and compare survival rates among low and high *DNMT3B7* expression groups.

## Results

### *DNMT3B7* expression is up-regulated in a majority of patient tumor samples

In order to determine the role of *DNMT3B7* in tumor progression across 33 cancer types, all available data from the GDC were downloaded and analyzed for expression of the 94bp intron sequence specific to *DNMT3B7*. Table 1 shows the complete results of our analysis, with statistical significance indicated where appropriate.

**Table 1:** Results of *DNMT3B7* expression across all cancer types.

Cancer Name	GDC Name	Sample Size	Normal vs. Tumor	Matched Normal vs. Tumor	Stage	Survival
Adrenocortical Carcinoma	ACC	79	NA	NA	No	No
Bladder Urothelial Carcinoma	BLCA	430	Yes (p<0.001)	Yes (p<0.001)	No	No
Breast Invasive Carcinoma	BRCA	1097	Yes (p<0.001) [10]	Yes (p<0.001) [10]	Yes (p = 0.01) [10]	No (p = 0.053)
Cervical Squamous Cell Carcinoma and Endocervical Adenocarcinoma	CESC	309	Yes (p = 0.022)	No	NA	Yes (p = 0.025)
Cholangiocarcinoma	CHOL	45	No	NA	No	No
Colon Adenocarcinoma	COAD	334	Yes (p<0.001)	Yes (p<0.001)	No	No
Lymphoid Neoplasm Diffuse B cell Lymphoma	DLBC	48	NA	NA	NA	No
Esophageal Carcinoma	ESCA	196	Yes (p<0.001)	Yes (p = 0.001)	Yes (p = 0.012)	No
Glioblastoma Multiforme	GBM	168	NA (No, primary vs recurrent)	NA (No, primary vs recurrent)	NA	No
Head/Neck Squamous Cell Carcinoma	HNSC	566	Yes (p<0.001)	Yes (p<0.001)	No	No
Kidney Chromophobe	KICH	91	Yes (p<0.001)^	No	No	No
Kidney Renal Clear Cell Carcinoma	KIRC	612	Yes (p = 0.023)	No	Yes (p = 0.010)	Yes (p = 0.009)
Kidney Renal Papillary Cell Carcinoma	KIRP	323	Yes (p<0.001)	Yes (p<0.001)	Yes (p = 0.02)	No
Acute Myeloid Leukemia	LAML	173	NA	NA	Yes (p<0.001)	Yes (p = 0.035)
Brain Lower Grade Glioma	LGG	541	NA (Yes, primary vs recurrent, p = 0.005)	NA (No, primary vs recurrent)	NA	No (p = 0.054)
Liver Hepatocellular Carcinoma	LIHC	424	Yes (p<0.001)	Yes (p<0.001)	No	No
Lung Adenocarcinoma	LUAD	576	Yes (p<0.001)	Yes (p<0.001)	No	No
Lung Squamous Cell Carcinoma	LUSC	553	Yes (p<0.001)	Yes (p<0.001)	Yes (p = 0.003)	No
Mesothelioma	MESO	87	NA	NA	No	Yes (p = 0.013)
Ovarian Serous Cystadenocarcinoma	OV	309	NA (No, primary vs recurrent)	NA (No, primary vs recurrent)	NA	No
Pancreatic Adenocarcinoma	PAAD	183	No	No	No	No
Pheochromocytoma and Paraganglioma	PCPG	185	No	No	NA	No
Prostate Adenocarcinoma	PRAD	558	No	No	NA	No
Rectum Adenocarcinoma	READ	105	Yes (p<0.001)	NA	No	No
Sarcoma	SARC	266	No	No	NA	Yes (p = 0.003)
Skin Cutaneous Melanoma	SKCM	473	NA	NA	No	Yes (p = 0.003)
Stomach Adenocarcinoma	STAD	450	Yes (p<0.001)	Yes (p<0.001)	No	No
Testicular Germ Cell Tumors	TGCT	154	NA	NA	Yes (p<0.001)	No
Thryoid Cancer	THCA	572	Yes (p<0.001)^	Yes (p<0.001)^	No	No
Thymoma	THYM	122	NA	NA	NA	No
Uterine Corpus Endometrial Carcinoma	UCEC	198	Yes (p<0.001)	Yes (p = 0.014)	NA	No
Uterine Carcinosarcoma	UCS	57	NA	NA	NA	No
Uveal Melanoma	UVM	80	NA	NA	No	No

Normal vs. Tumor includes all patient samples available while Matched Normal vs. Tumor only includes matched samples.

“Yes” indicates data are statistically significant with increased *DNMT3B7* expression observed in tumor samples, higher stage, and/or poor survival groups.

“No” indicates data were not statistically significant.

“NA” indicates data were not available so analysis could not be completed.

^ indicates that the pattern was altered and *DNMT3B7* expression was higher in normal samples compared to tumor tissues for that sample.

**Fig 1:**
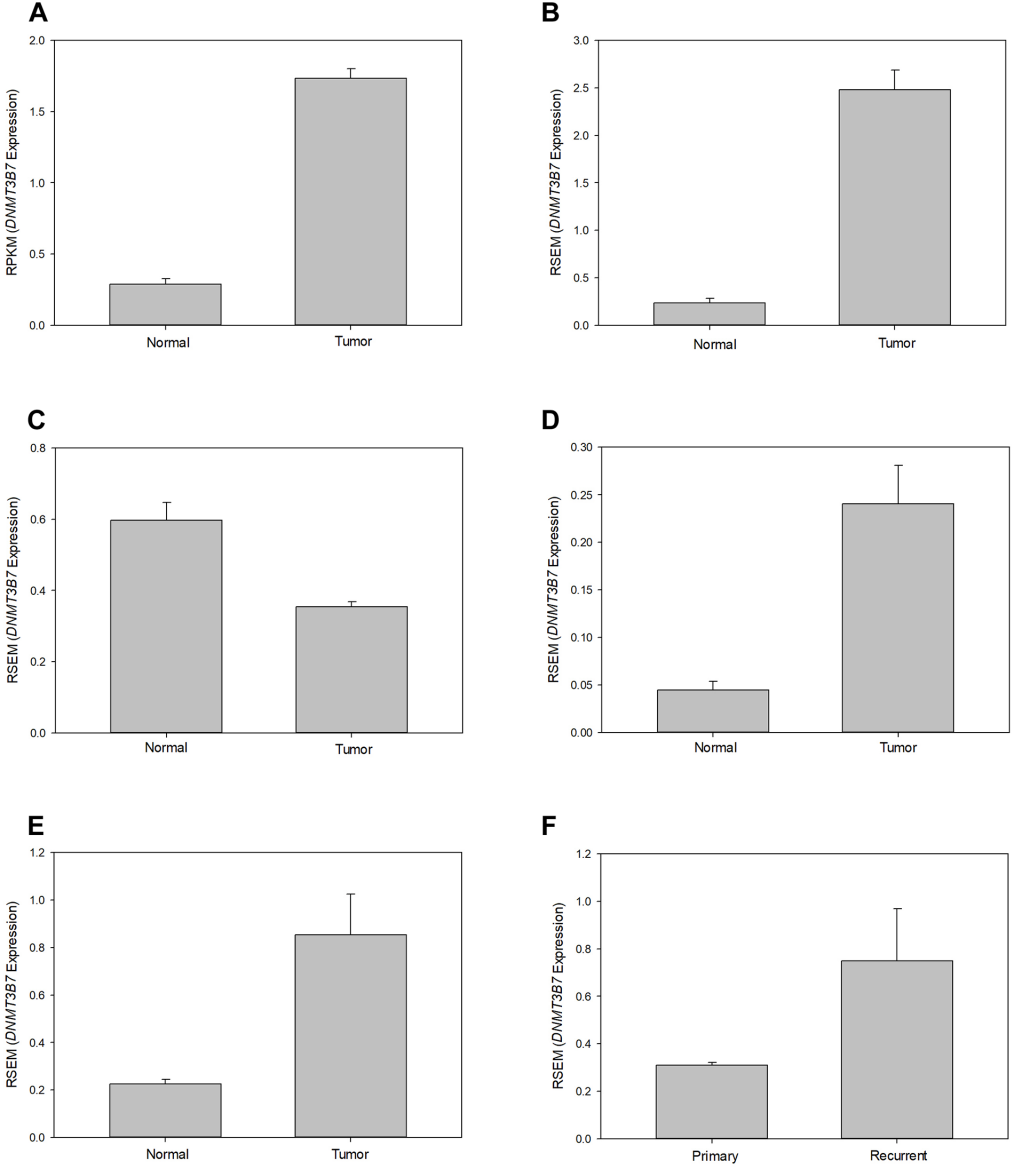
Expression of *DNMT3B7* in normal and tumor patient samples. Representative graphs of 6 different tumor samples showing relative *DNMT3B7* expression, as measured by reads per kilobase per million (RPKM) or RNA-Seq by Expectation Maximization (RSEM), in unmatched normal and tumor tissues in (A) HNSC, (B) UCEC, and (C) THCA. Expression of *DNMT3B7* in matched patient samples is shown in (D) LIHC and (E) LUAD. *DNMT3B7* expression in primary and recurrent tissues in (F) LGG (*p* = 0.005) was assessed when normal samples were not available. All samples shown here were significant, *p* < 0.001, unless otherwise stated.

Of the 21 samples in which both normal and tumor tissues were available, 14 (67%) showed increased expression of *DNMT3B7* in tumor samples compared to normal tissue, while two—KICH and THCA—showed decreased expression in tumor samples compared to normal (Fig 1A–1C). Of the 16 tumor types that showed significant differences in expression between normal and tumor tissue, 11 had similar patterns in matched patient samples, indicating that these results were not due to outliers in the group but rather genetic changes occurring in patients as their tumor developed and progressed (Fig 1D and 1E). Finally, while 12 of the tumor types did not have normal tissues available for analysis, three of these—GBM, LGG, and OV—did have primary and recurrent tissue samples which were utilized for comparison. Of these three tumor types, LGG showed increased expression in recurrent tumors compared to primary tumors (Fig 1F).

### *DNMT3B7* expression correlates to increased stage in some cancers

**Fig 2:**
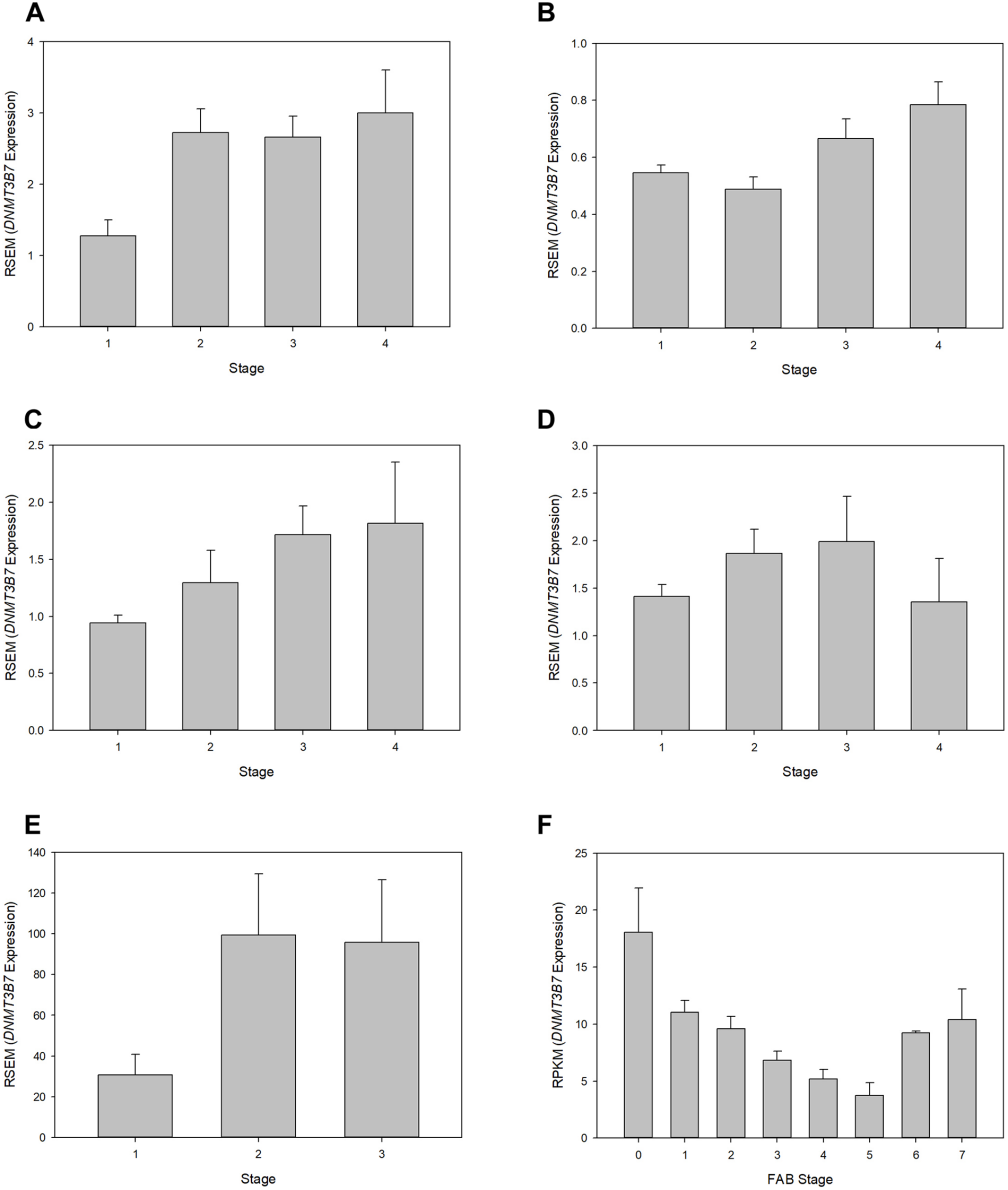
Relative *DNMT3B7* expression correlates to clinical staging. *DNMT3B7* expression was compared to clinical stage and shown to be significantly different in (A) ESCA, *p* = 0.012; (B) KIRC, *p* = 0.010; (C) KIRP, *p* = 0.02; (D) LUSC, *p* = 0.003; (E) TGCT, *p*<0.001; and (F) LAML, *p*<0.001. For (E) TGCT, there were no patient samples with a stage IV diagnosis. (F) LAML staging was measured using the French-American-British (FAB) classifications.

In order to further assess the effects of *DNMT3B7* expression on clinical tumor progression, an analysis of expression based on diagnostic staging was completed. Of the 22 tumor samples for which data were available, 7 (32%) showed changes in expression relative to stage. In almost all cases, *DNMT3B7* expression increased as clinical stage advanced indicating that *DNMT3B7* expression correlates with tumor progression as measured by stage. Fig 2 shows the results of every cancer type with significant results except for BRCA, for which these data were published previously by our laboratory [10].

### High expression of *DNMT3B7* correlates with poorer survival in six tumor types

**Fig 3:**
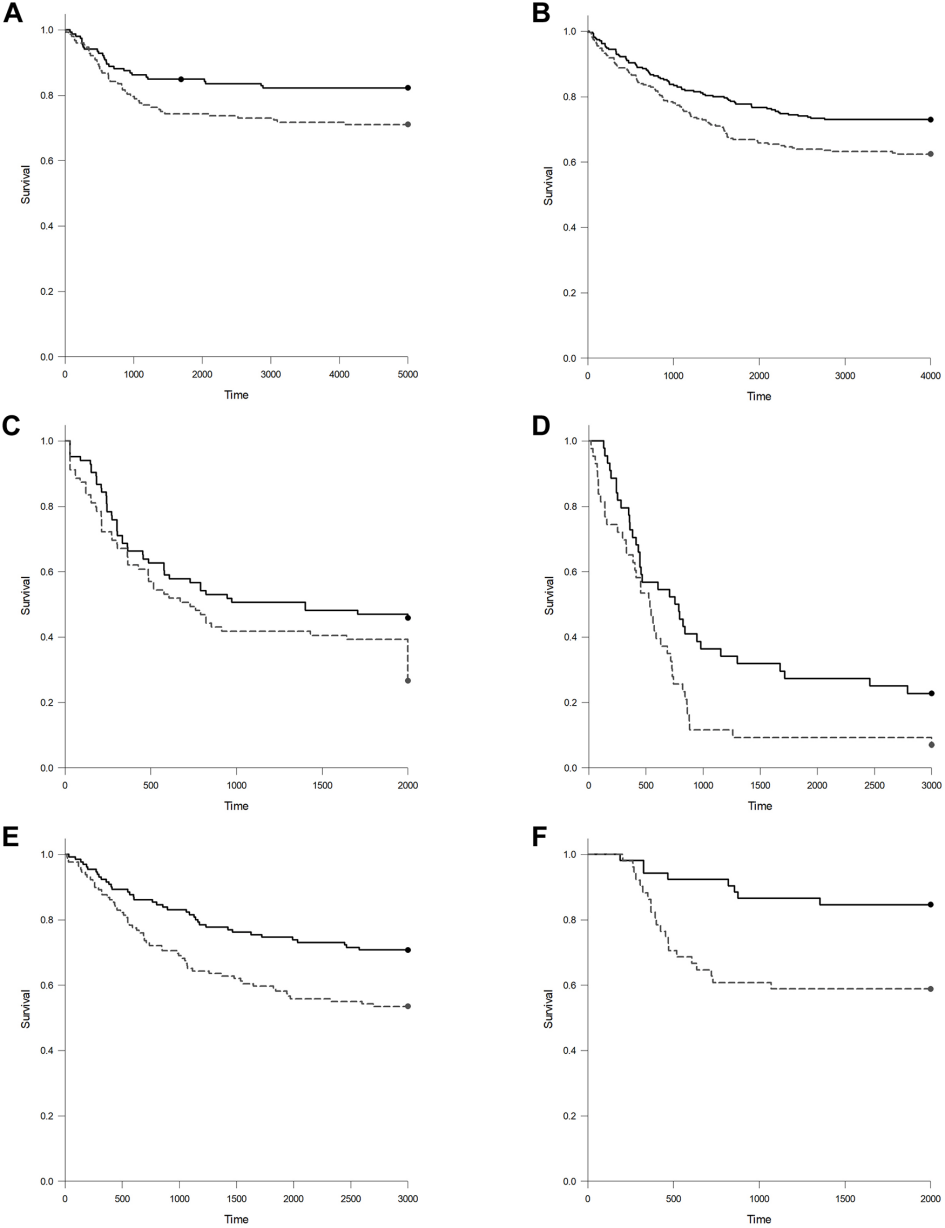
Patients with high levels of *DNMT3B7* expression have lower survival rates than those with low expression levels. The median *DNMT3B7* expression for each individual tumor was determined to divide patients with that tumor into “high” (gray, dotted line) and “low” (black, solid line) expression groups. Kaplan-Meier curves were generated and statistical significance was determined for (A) CESC, *p* = 0.025; (B) KIRC, *p* = 0.009; (C) LAML, *p* = 0.035; (D) MESO, *p* = 0.013; (E) SARC, *p* = 0.003; and (F) SKCM, *p* = 0.003.

A final analysis was completed to determine the effect of high *DNMT3B7* expression on patient survival. While survival is not a direct measure of tumor progression, due to the availability of therapeutics for specific malignancies, ability to diagnose some tumors at early stages, etc., we thought it was important to determine if there was any relationship between *DNMT3B7* and survival as part of this analysis. Therefore, patients in each cancer type were divided into a “high” and “low” expression group based on the median *DNMT3B7* expression for that cancer. A Kaplan-Meier analysis was completed and significant results are shown in Fig 3. Of the 33 cancers tested, only 6 (18%) showed a significant change in survival. In all cases, patients with high expression of *DNMT3B7* had lower survival rates than those with low expression.

## Discussion

Due to the heterogeneity of tumors, it is rare to find one gene that is mutated across many cancer types that can be targeted by therapeutics. Furthermore, in the rare cases where a gene is mutated in many cancers, (e.g. p53), the mutations are often in different parts of the gene making it impossible to develop one therapeutic that is useful for all patients. However, the results shown here indicate that *DNMT3B7* may be a useful target across tumors in the future. Because of the unique sequence it retains of 94bp of intron 10 leading to a premature stop codon, there is a potential for specific targeting of this protein in cells. Our analysis here utilized that unique sequence as a measure of *DNMT3B7* expression in all available patient samples on the GDC and showed that this sequence is correlated to tumor progression across all types of cancers—including carcinomas, sarcomas, and leukemias—as measured by tissue type, clinical stage, and survival across many cancer types.

Of the 33 cancers with data available on GDC, 22 (67%) showed significant effects of *DNMT3B7* expression with relation to at least one of our measurements presented here. Of the 11 tumor types for which *DNMT3B7* expression did not show any effect, five (DLBC, GBM, OV, THYM, and UCS) had no data available to assess anything except survival (Table 1). Therefore, it is possible that *DNMT3B7* expression may have an effect in these cancers also, but we are unable to determine that based on the data available at this time. Furthermore, 7 cancers had sample sizes under 100 patients which may have prevented statistical significance from being achieved.

It is always important to determine if the experimental results previously shown *in vitro* and *in vivo* match those seen in clinical samples. Overall, we observed that the results shown by Ostler and colleagues [7] in which *DNMT3B7* is expressed in virtually all (84%) cancer cell lines tested is confirmed here to a similar degree. Based on the data available on GDC, we were able to show altered expression of *DNMT3B7* in 16 out of 21 samples (74%). Furthermore, we see that the previously published *in vitro* work in breast cancer matches with our clinical analysis (Table 1 and [10]). Shah and colleagues showed that DNMT3B7 expression led to lymphomagenesis [9], however these results could not be recapitulated due to the lack of available data on GDC. Finally, it may have been hypothesized that, based on the results of Ostler and colleagues in neuroblastoma [11], that the brain tumors examined here (GBM and LGG) would have shown a flipped pattern of expression in which higher *DNMT3B7* expression correlated with normal samples compared to tumor samples. Unfortunately, normal samples were not available for this analysis, so we are unable to confirm or deny that hypothesis. We did observe that LGG showed increased expression of *DNMT3B7* in recurrent tumors compared to primary tumors (Table 1 and Fig 1F) which would oppose the results seen in neuroblastoma. However, this is not overly surprising since neuroblastoma is a pediatric cancer that is caused by problems in early development and neurogenesis while LGG is diagnosed in adult brains and, therefore, is caused by entirely different mechanisms [14].

Our analysis of differences in expression based on clinical stage, while providing some useful data (Fig 2), was not as informative as hoped based on a few factors. First, we were unable to analyze 33% of our total samples due to a lack of available clinical data for our patient samples. It is quite possible that significant changes in *DNMT3B7* expression correlate with stage in more cancers than shown here, but we cannot determine that based on the data available. Next, because many of the cancers had relatively small sample sizes to begin with, this was then further exacerbated by the fact that we had to subdivide these samples into smaller groups in order to complete our analysis. Therefore, it is once again possible that *DNMT3B7* may play a larger role than observed here, but differences in sample size do not allow us to observe these trends at this time. Additionally, analysis of the effects on clinical stage is dependent on having enough patient samples diagnosed at each stage, which was not always possible. As shown in Fig 2E, some cancers did not have any patients diagnosed at one stage or another, which affected our results. In other cases, such as PAAD, of the 183 patient samples available, 21 were stage I, 151 were stage II, 3 were stage III, and 5 were stage IV. Small sample sizes in all but stage II led to difficulties in achieving any sort of sound statistical analysis. LAML had to be divided based on 8 FAB classifications which led to many more divisions than other tumor types. While our results did obtain statistical significance (Fig 2F), there was no distinguishable pattern except in the most advanced stages (6 and 7).

As stated previously, survival is not a direct measure of tumor progression and it can be affected by many factors including ability to diagnose early and available treatments. However, it is certainly the most important clinical outcome for patients and their families and, for that reason, it was included as part of our analysis (Fig 3). We saw significant differences in survival rates in LAML, which is the tumor type in which *DNMT3B7* was originally identified [7]. Conversely, we did not see a significant difference in survival in BRCA (*p* = 0.053), even with a large sample size and previous *in vitro* data indicating a role in tumor progression [10]. However, this could be due to the fact that breast cancer is typically diagnosed at an early stage and has many good treatment options available, leading to increased survival rates compared to other cancers.

Our results show that no one cancer had statistically significant results in all three categories tested (normal versus tumor tissue, stage, and survival). However, 7 tumor types (BRCA, CESC, ESCA, KIRC, KIRP, LAML, and LUSC; Table 1) had changes in two different categories, assuming expression in normal and tumor samples in matched and unmatched tissues are considered one category. Because previous *in vitro* studies in LAML and BRCA have already been completed and confirmed [7, 10], these results suggest that the other 5 cancers listed above should be prioritized for future studies involving DNMT3B7. Specifically, continued *in vitro* and *in vivo* work, matched with clinical samples, is imperative to elucidate the functional role of DNMT3B7 in cancer and strengthen the likelihood of therapeutic targeting of this aberrant protein across cancer types in the future. Taken together, the results presented here demonstrate that *DNMT3B7* has a role in tumor progression across cancers of all types and is a promising target for future drug development.
